# Needle tract seeding and abdominal recurrence following pre-treatment biopsy of gastrointestinal stromal tumors (GIST): results of a systematic review

**DOI:** 10.1186/s12893-022-01648-2

**Published:** 2022-05-21

**Authors:** Jens Jakob, Rashad Salameh, David Wichmann, Nicos Charalambous, Anne-Christine Zygmunt, Inga Kreisel, Judith Heinz, Michael Ghadimi, Ulrich Ronellenfitsch

**Affiliations:** 1 Department of Surgery, Sarcoma Unit, University Medical Center Mannheim, Th.-Kutzer-Ufer 1–3, 68163 Mannheim, Germany; 2 Department of Visceral, Thoracic, Vascular and Transplant Surgery, University Hospital Dresden, Dresden, Germany; 3 Department of General, Visceral and Pediatric Surgery, University Medical Center, Goettingen, Germany; 4 Department of Medical Statistics, University Medical Center Goettingen, Goettingen, Germany; 5 Department of Surgery, University Hospital Halle, Halle, Germany

**Keywords:** Core needle biopsy, Fine needle aspiration, FNA, Preoperative biopsy, Metastasis, GIST

## Abstract

**Background:**

Gastrointestinal stromal tumors (GIST) are rare abdominal tumors. Pretreatment biopsies may be used to diagnose a GIST and enable tailored treatment. Some experts are skeptical about biopsies because they fear tumor cell seeding. The objective of this study was to determine if pretreatment biopsy is associated with increased tumor recurrence.

**Methods:**

We performed a systematic literature search and included studies assessing the oncological outcome of GIST patients who underwent a pre-treatment core needle biopsy or fine needle aspiration. We assessed methodological quality with the Newcastle-Ottawa-Scale for non-randomized studies. This review was registered in the PROSPERO database (CRD42021170290).

**Results:**

Three non-randomized studies and eight case reports comprising 350 patients were eligible for inclusion. No prospective study designed to answer the review question was found. One case of needle tract seeding after percutaneous core needle biopsy of GIST was reported. None of the studies reported an increased rate of abdominal recurrence in patients with pretreatment biopsy.

**Conclusions:**

The existing evidence does not indicate a relevant risk of needle tract seeding or abdominal recurrence after pre-treatment biopsy of GIST. Biopsy can safely be done to differentiate GIST from other tumors and to select the most appropriate treatment.

**Supplementary information:**

The online version contains supplementary material available at 10.1186/s12893-022-01648-2.

## Introduction

Gastrointestinal stromal tumor (GIST) is the most frequent mesenchymal malignancy of the gastrointestinal tract [1]. The estimated worldwide incidence is 10–15 per million per year [2]. Most GIST are characterized by a gain of function mutation of c-kit and the platelet derived growth factor receptor (PDGFR) [3, 4]. Prognosis of locally advanced or metastatic GIST has improved remarkably after introduction of targeted therapy with receptor tyrosine kinase inhibitors (RTKI) [5–7].

The cornerstone of successful treatment of localized GIST is complete resection [8–10]. In locally advanced cases, preoperative treatment with the RTKI imatinib frequently leads to pronounced tumor response and reduced extent of the operation and surgical morbidity [11–15]. A prerequisite of imatinib efficacy is pre-treatment biopsy yielding proof of a sensitive mutation in c-kit or PDGFR. About 20–30% of all GIST are lacking KIT/PDGFRA mutations; these tumors may yield defects in the succinate dehydrogenase (SDH) complex.[16] They may respond to treatment with other RTKI than imatinib. Nevertheless, a neoadjuvant treatment approach is not recommended in these cases.

Small GIST without mitotic activity (e.g. gastric GIST of less than 2 cm size) is frequently found as incidentaloma [17, 18]. Its prognosis is excellent and usually a watch-and-wait strategy is sufficient. A biopsy proving GIST and pointing towards a low risk of recurrence is helpful in decision-making in these cases. Many other GISTs are suspected after cross sectional imaging or endoscopy indicated for (occult) gastrointestinal bleeding, pain, or gastrointestinal passage disorders [19]. Differential diagnoses are benign lesions but also lymphoma, neuroendocrine carcinoma, or other sarcoma subtypes [20–22]. Multimodal treatment of these tumors is different and surgical strategies vary in those patients who require resection (e.g., lymphadenectomy, e.g., dimension of resection margins etc.) [23–25].

In summary, pre-treatment biopsies may help to guide treatment decisions and improve shared decision making in GIST patients. However, some experts are skeptical about biopsies because they fear tumor seeding and increased recurrence rates. Needle tract seeding has in fact been reported for various other abdominal tumors [26–30]. However, the incidence of needle tract seeding after pre-treatment biopsy of GIST remains unknown. Furthermore, current NCCN, ESMO and UK guidelines to not cite prospective studies evaluating the risk of recurrence after pretreatment biopsy considering different biopsy techniques, adjuvant treatment and tumor-associated risk of recurrence[8–10]. We therefore initiated this systematic review to evaluate the rate of abdominal wall and peritoneal recurrences after pre-treatment biopsy of GIST.

## Materials and methods

This review was conducted according to the Preferred Reporting Items for Systematic Reviews and Meta-Analyses (PRISMA) and registered in the PROSPERO database, an international register of systematic reviews (register number CRD42021170290). The registration is accessible online (https://www.crd.york.ac.uk/prospero/display_record.php?ID=CRD42021170290). The PRISMA checklist is provided in the Additional file 1. No financial or non-financial support was given for the review.

### Review question and eligibility criteria

The primary review question was evaluating the risk of needle tract seeding and recurrence after pretreatment biopsy of suspected GIST. Studies evaluating adult patients (18 years or older) with suspected GIST were included. Studies with at least one patient with suspected GIST and pre-treatment biopsy were included. Studies without information on oncological outcome (e.g., survival, rate, or number of local or distant recurrences, rate or number of needle tract seeding) were excluded. Assumed influencing factors such as biopsy technique, adjuvant treatment and tumor-associated risk of recurrence were documented and analyzed. Eligible studies were searched for the outcome of patients who did not undergo pre-treatment biopsies for comparison.

### Information sources and search strategy

As of 3rd of November 2021, the PubMed database was searched for eligible studies (search strategy in Table 1). Additionally, the bibliographies of the included studies were hand-searched for eligible references. Moreover, data and references from the NCCN, UK and ESMO guideline for diagnosis and treatment of GIST were searched [8–10]. Prospective and retrospective studies of any design as well as case reports and reviews were included. Publications in English and German-language were included into the analysis. No restrictions were made for publication date.

**Table 1:** Search strategy

Search #	Title	Terms	Hits
1	GIST	“gastrointestinal”[Title/Abstract] AND “stromal”[Title/Abstract] AND (“tumor”[Title/Abstract] OR “tumors”[Title/Abstract] OR “neoplasm”[Title/Abstract] OR “neoplasms”[Title/Abstract] OR “sarcoma”[Title/Abstract] OR “sarcomas”[Title/Abstract] OR “cancer” [Title/Abstract] OR “cancers”[Title/Abstract])	9760
2	Biopsy	“biop*“[Title/Abstract] OR “fine needle“[Title/Abstract] OR “core needle“[Title/Abstract]	569,221
3		#1 AND #2	877
4	Survival	survival[Title/Abstract] OR mortality[Title/Abstract] OR death[Title/Abstract]	2,293,766
5	Recurrence	“recurrence“[Title/Abstract]	321,063
6	Seeding	“seeding“[Title/Abstract] OR “metastasis“[Title/Abstract] OR “metastases“[Title/Abstract]	406,051
7		#4 OR #5 OR #6	2,748,749
8		#3 AND #7	264
9		#3 AND #7 Filters: Humans	205
10		#3 AND #7 Filters: Humans; English	150

### Study selection and data extraction

Two investigators (JJ, RS) reviewed all selected abstracts independently. Disagreement was resolved by consensus. Data were extracted from the full text articles of the selected abstracts independently by two investigators (JJ, RS). If available, the following prespecified data were extracted: name of first author and year of publication, type of study, number of patients, number of patients with GIST, tumor associated risk of recurrence, number of patients with GIST undergoing pre-treatment biopsy, type of biopsy (percutaneous core needle biopsy (CNB) vs. percutaneous fine needle aspiration (p-FNA) vs. endoscopic ultrasound guided fine needle aspiration (EUS-FNA)), number of patients with adjuvant imatinib treatment, number of patients with seeding along the biopsy tract, number of patients with recurrence, number of patients with recurrence possibly associated with pre-treatment biopsy, number or procedure associated complications, accuracy/sensitivity/specificity of pre-treatment biopsy.

### Assessment of methodological quality

Assessment of methodological quality was done at study level. Since most studies were expected to be cohort studies, we performed assessment of methodological quality with the Newcastle-Ottawa-Scale (NOS) [31]. The NOS contains eight items for cohort studies, categorized into three dimensions: selection, comparability, and outcome. Studies that received a score of seven or above were considered as high quality. Three reviewers (RS, UR, JJ) performed quality assessment. Disagreements were resolved by discussion and consensus.

### Statistical analysis

Data are presented descriptively as numbers of patients, median or mean and range. Due to the heterogeneous study designs and the poor data availability a quantitative data synthesis and analysis was not feasible.

## Results

**Fig. 1:**
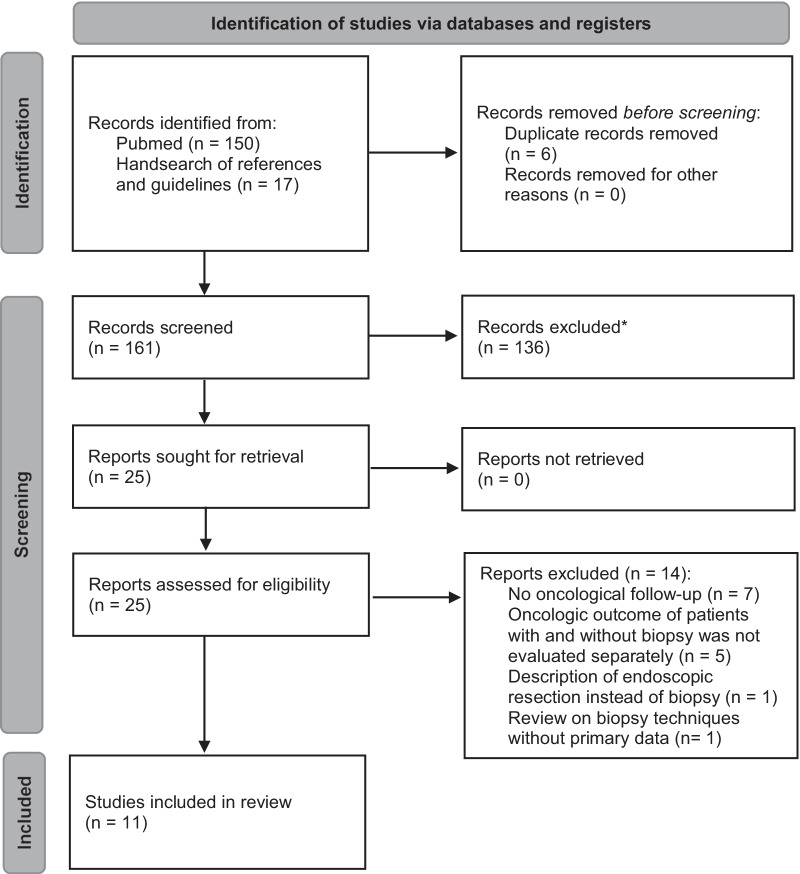
Prisma 2020 flow diagram. *No automation tool was used in this review to exclude or include reports into the review

The database searches produced 150 articles (Fig. 1: PRISMA flow diagram). Seventeen additional articles were identified through retrieval and search of the references and the NCCN, UK and ESMO guidelines [8–10]. Twenty-five full-text articles were assessed for eligibility. Fourteen full text articles were excluded from the review [32–45]. Excluded manuscripts either reported technical aspects of but not oncological follow up after GIST biopsies (n = 7), described cases of endoscopic resection instead of biopsy (n = 1), summarized the oncologic outcome of patients with and without biopsy without addressing who had more recurrences (n = 5) or comprised reviews on biopsy techniques without presenting primary data (n = 1).

The literature review did not identify any prospective study that evaluated the association between biopsy and recurrence in GIST. Besides eight case reports [46–53], three retrospective studies [54–56] were included in the review (compare Table 2): Akahoshi et al. evaluated the oncological outcome of patients with small gastric GIST who had undergone pretreatment EUS-FNA prior to tumor resection [56]. The authors reported correctness of the pretherapeutic biopsies in 32 of 44 cases (73%), and there were no biopsy-associated complications and no recurrences. The authors concluded that pretreatment EUS-FNA is safe and efficient in small gastric GIST. Eriksson et al. performed a post hoc analysis of the SSG/AIO adjuvant trial for GIST [55]. All patients had high risk GIST and all patients received adjuvant imatinib. Forty-seven of the 389 patients in the study had undergone a pretherapeutic biopsy (CNB or FNA or both). At a median follow-up of 54 months, there was no difference in tumor-free survival as a function of biopsy. In subgroup analysis, recurrence free survival was improved in patients with GIST larger than 10 cm who had undergone pretreatment biopsies. The authors concluded that pretreatment biopsy of a suspected GIST may not increase the risk for recurrence in patients who receive adjuvant imatinib after the biopsy. Houdt et al. evaluated prospective GIST databases from two referral centers [54]. They included 220 patients in the analysis, 186 of whom had received a pretherapeutic biopsy (CNB, FNA or both). In multivariate analysis adjusting for tumor and treatment-associated factors (including adjuvant imatinib and risk of recurrence), pretherapeutic biopsy did not increase the recurrence rate. Of the 186 patients with biopsy, one patient had a biopsy-associated local recurrence after percutaneous core needle biopsy; comparison of computed tomography scans during the biopsy and at the time of recurrence show that the local recurrence was located immediately in the needle tract of the biopsy.

**Table 2:** Details of the selected studies

First author and year of publication	Houdt 2021 [54]	Akahoshi 2014 [56]	Eriksson 2016 [55]
Study type	Retrospective analysis of two prospectively kept databases	Retrospective analysis of one prospectively kept database	Posthoc analysis of a randomized trial
Aim of the study	to evaluate local recurrence free survival and disease specific survival of GIST patients with or without pretreatment biopsy	To define diagnostic accuracy and safety of and tumor recurrence after EUS-FNA of gastric GIST	To evaluate if percutaneous tumor biopsy has an impact on RFS and OS in patients with GIST receiving adjuvant imatinib after tumor resection
Number of patients	228	44	389
Risk of recurrence (low, intermediate, high)	86, 43, 80, (19 unknown)	33, 10, 0	0, 0, 389
Patients with (neo-) adjuvant imatinib	Neoadjuvant 100 adjuvant 158	0	Adjuvant 389
Patients with biopsy	186	44	47
Biopsy technique	CNB = 166, FNA = 20, Transcutaneous = 70, Endoluminal = 116	EUS-FNA = 44	CNB = 33, FNA = 22, CNB and FNA = 8
Complications of biopsy	N/A	0	N/A
Number of correct diagnoses from pretreatment biopsies	N/A	32	N/A
Recurrence related to biopsy	1	0	N/A^b^
Total number of abdominal wall or peritoneal recurrences	25	0	N/A^b^
Follow-up (months)	Median = 53	Mean = 35, range = 2–108	Median = 54^a^

^a^Median follow-up was not reported in the manuscript of Eriksson et al. We report median follow-up as reported in the primary publication of the trial [55]

^b^Only recurrence free survival (RFS) and overall survival (OS) presented

Eight case reports describe surgical or multimodal treatment of GIST after pre-treatment biopsy. Tumor recurrence was not reported in any of the case reports (Table 3). No case report of needle tract seeding after pretreatment biopsy of GIST was published.

**Table 3:** Details of the selected case reports

First author and year of publication	Cecka 2011	Min Wang 2020	Suzuki 2011	Wollina 2015	Kane 2019	Nakamura 2012	Yin 2018	Zhang 2021
Risk of recurrence (low, intermediate, high)	High risk	High risk	High risk	Intermediate risk	Intermediate risk	Intermediate risk	High risk	High risk
Adjuvant imatinib	0	0	1	1	1	1	1	1
Patients with biopsy	1	1	1	1	1	1	1	1
Complications of biopsy	N/A	N/A	N/A	N/A	N/A	N/A	N/A	0
Number of correct diagnoses from pretreatment biopsies	1	1	1	1	1	1	1	1
Recurrence related to biopsy	0	0	0	0	0	0	0	0
Total number of abdominal wall or peritoneal recurrences	0	0	0	0	0	0	0	0
Follow-up (months)	66	12	14	8	18	30	48	20

Assessment of methodological quality using the Newcastle-Ottawa Scale is displayed in Table 4. The overall methodological quality of studies revealed that three studies were of high quality. One study was of moderate quality because of a lack of comparability.

**Table 4:** Assessment of methodological quality with the Newcastle-Ottawa-Scale

Publication (first author, year)	Selection (up to ****)	Comparability (up to **)	Outcome (up to ***)
Eriksson 2016 [55]	_****_	_**_	_***_
Akahoshi 2014 [56]	_***_		_**_
Houdt 2021 [54]	_****_	_**_	_***_

## Discussion

A pre-treatment histology is the prerequisite and cornerstone of shared decision making and multimodal treatment considerations. There is strong evidence of a survival advantage for patients who were treated according to multidisciplinary tumor board decisions before surgery and who had surgery at expert centers [57–59]. Nevertheless, the risk of tumor cell dissemination is frequently discussed in the context of GIST biopsies. The current NCCN guideline acknowledges these concerns and considers endoscopic biopsy to be advantageous over percutaneous biopsy due to the assumed lower risk of intra-abdominal tumor dissemination [8]. Therefore, the primary question of this systematic review was whether there is evidence for an increased risk of recurrence of needle tract seeding after pretreatment biopsy of GIST. As main results, this systematic review revealed that the association of biopsy and recurrence of GIST was never evaluated in a prospective trial, that only one case of needle tract seeding has been reported so far, and that no study reported increased recurrence rates after pretreatment GIST biopsy.

In other malignancies, systematic reviews and meta-analyses point towards a certain risk of needle tract seeding. The highest risk is reported for hepatocellular carcinoma (HCC) with 2.7% [60]. The estimated incidence of needle tract seeding in other tumors is much lower than 1% [61, 62]. The estimated incidence in other soft tissue sarcomas was evaluated in a pooled analysis of four cohorts evaluating 547 patients with retroperitoneal sarcomas [63]. The authors reported two cases of needle tract seeding resulting in an estimated incidence of 0.37% that is comparable to the results of this review.

Apart from tumor biology, the biopsy technique itself may influence the risk of needle tract seeding and recurrence. Regarding biopsy route and technique, percutaneous core needle biopsies may have the highest risk for needle tract seeding. In concordance, the only case of needle tract seeding in our review was documented after percutaneous CNB – if a coaxial sheathed biopsy needle was used as recommended was not reported [54, 64]. We did also include cases with endoscopic fine needle aspirations in this analysis for two reasons: First, there were case reports of bleeding into the abdominal cavity after EUS-FNA, and we assume that if bleeding into the abdominal cavity occurs, the same might be true for tumor seeding [65, 66]. There was one cohort study reporting oncological results after EUS-FNA of small GIST without any recurrence [56]. Although the risk of recurrence in small gastric GIST is low or very low in general, we still think this is an important piece of information since a single recurrence would be a very strong indicator of a clinically relevant tumor cell dissemination after EUS-FNA in small gastric GIST. Thus, EUS-FNA may be safely used in these tumors to confirm the diagnosis or differentiate these lesions from other gastric tumors.

Tumor risk of recurrence and the administration of adjuvant treatment may have an impact on the incidence of tumor growth after biopsy-related tumor cell dissemination. Both factors are related to each other. Patients with intermediate or high risk GIST frequently receive adjuvant treatment [8–10]. According to the post-hoc analysis of the SSG-AIO adjuvant trial, these patients do not have an increased risk of recurrence [55]. In general, the data identified and summarized in this review are not sufficient to stratify the risk of recurrence after biopsy according to general risk of recurrence and administration of adjuvant treatment. If preoperative imatinib treatment may lead to less invasive surgery and organ preservation in intermediate or high risk GIST, patients are recommended to undergo a biopsy to enable tailored treatment [8–10]. Several studies are available on neoadjuvant treatment of GIST [11–15, 67–70]. We assumed that biopsies had been taken before preoperative treatment and screened the full text manuscripts. None of the studies explicitly discussed the methodology of pre-therapeutic biopsies. Neither described a case of needle tract seeding.

It arises the question if those who receive surgery as the only treatment are exposed to an increased risk of recurrence after pretreatment biopsy. For small GIST, there does not seem to be any increased risk (see above). Yet, it is one limitation of this review that we cannot present data for patients with intermediate or high risk GIST who underwent surgery alone. Upfront resection may be an alternative provided that resection morbidity is low (e.g. laparoscopic gastric wedge resection) and other histologies have been taken into account (e.g. lymphoma, neuroendocrine tumors).

This systematic review has limitations. No prospective study was found which was designed to answer the review question with its primary endpoint. Only retrospective studies, post hoc analyses and case reports were available. This systematic review relied on a limited number of databases for the identification of potentially eligible studies. The included studies lack clear correlations or information on the risk of metastasis, adjuvant therapy, type of biopsy as well as localization of tumor recurrence. Due to the limited and heterogeneous data, no statistical analysis in the sense of a meta-analysis or a subgroup analysis was possible. The total number of patients included is small and we only included GIST patients and no patients with other abdominal tumors. These limitations may be overcome by conducting a well-designed randomized prospective trial evaluating the risks and benefits of pretreatment biopsies in general. Such a prospective trial should comprise not only GIST patients but patients with any abdominal mass suspected to be a malignant tumor. It would require thorough statistical preparation taking into account the incidence of various tumors, the relevance of preoperative treatment and the known or estimated risk of needle tract seeding. Despite these limitations, the results of the present literature review and evidence synthesis may help patients and physicians decide whether to perform a biopsy in the presence of an abdominal mass.

## Conclusions

There is a strong rationale to perform pretreatment biopsies in GIST. Histological proof of GIST enables shared decision making and multimodal treatment according to guidelines after discussion in multidisciplinary tumor boards – ultimately leading to potentially improved survival. This systematic literature review revealed no increased recurrence rates after EUS-FNA of low risk GIST treated by surgery and no increased recurrence rates after biopsy of intermediate and high risk GIST treated with combined medical and surgical treatment. For patients with intermediate- or high-risk GIST treated by surgery alone, this review contains only few data, and the safety of pretreatment biopsy cannot formally be proven. On the other hand, no cases of biopsy-associated recurrence were reported. In conclusion, the following pragmatic conclusions may be drawn from this systematic review: technically correctly performed GIST biopsies are safe and have a very low risk of needle tract seeding. Upfront resection as an alternative may be performed if resection morbidity is expected to be low (e.g. laparoscopic gastric wedge resection), other histologies (e.g. lymphoma, neuroendocrine tumors) are taken into account, and preoperative treatment is unlikely to decrease the extent of surgery.

## Supplementary information

**Additional file** **1: **The following supporting information can be downloaded: Prisma statements CRD42021170290.

## Data Availability

Further data are available from the corresponding author upon reasonable request.

## Abbreviations

CNB: Core needle biopsy
ESMO: European society of medical oncology
FNA: Fine needle aspiration
GIST: Gastrointestinal stromal tumor
NCCN: National comprehensive cancer network
NOS: Newcastle Ottawa Scale
RTKI: Receptor tyrosine kinase inhibitor
UK: United Kingdom
